# The Local South American Chicken Populations Are a Melting-Pot of Genomic Diversity

**DOI:** 10.3389/fgene.2019.01172

**Published:** 2019-11-19

**Authors:** Agusto Luzuriaga-Neira, Lucía Pérez-Pardal, Sean M. O’Rourke, Gustavo Villacís-Rivas, Freddy Cueva-Castillo, Galo Escudero-Sánchez, Juan Carlos Aguirre-Pabón, Amarilis Ulloa-Núñez, Makarena Rubilar-Quezada, Marcelo Vallinoto, Michael R. Miller, Albano Beja-Pereira

**Affiliations:** ^1^Centro de Investigação em Biodiversidade e Recursos Genéticos (CIBIO-InBIO), Universidade do Porto, Vairão, Portugal; ^2^Department of Animal Science, University of California, Davis, CA, United States; ^3^Centro De Biotecnología, Universidad Nacional de Loja, Loja, Ecuador; ^4^Universidad Nacional de Loja, Loja, Ecuador; ^5^Facultad de Ciencias Veterinarias, Universidad de Concepción, Chillán, Chile; ^6^Laboratório de Evolução (LEVO), Instituto de Estudos Costeiros (IECOS), Universidade Federal do Pará, Pará, Bragança, Brazil; ^7^Center for Watershed Sciences, University of California, Davis, CA, United States; ^8^Departamento de Geociências, Ambiente e Ordenamento do Território (DGAOT), Faculdade de Ciências, University of Porto, Porto, Portugal

## Abstract

Chicken have a considerable impact in South American rural household economy as a source of animal protein (eggs and meat) and a major role in cultural traditions (e.g., cockfighting, religious ceremonies, folklore). A large number of phenotypes and its heterogeneity are due to the multitude of environments (from arid to tropical rain forest and high altitude) and agricultural systems (highly industrialized to subsistence agriculture). This heterogeneity also represents the successive introduction of domestic chicken into this continent, which some consider predating Columbus’ arrival to South America. In this study, we have used next-generation restriction site-associated DNA sequencing to scan for genome-wide variation across 145 South American chickens representing local populations from six countries of South America (Colombia, Brazil, Ecuador, Peru, Bolivia, and Chile). After quality control, the genotypes of 122,801 single nucleotide polymorphisms (SNPs) were used to assess the genomic diversity and interpopulation genetic relationship between those populations and their potential sources. The estimated population genetic diversity displayed that the gamefowl has the least diverse population (θπ = 0.86; θS = 0.70). This population is also the most divergent (**F**_ST_ = 0.11) among the South American populations. The allele-sharing analysis and the admixture analysis revealed that the current diversity displayed by these populations resulted from multiple admixture events with a strong influence of the modern commercial egg-layer chicken (ranging between 44% and 79%). It also revealed an unknown genetic component that is mostly present in the Easter Island population that is also present in local chicken populations from the South American Pacific fringe.

## *Gallus gallus*, RADseq, population genetics, local resources, single nucleotide polymorphismsIntroduction

The domestic chicken, *Gallus gallus domesticus,* is a major source of animal protein (eggs and meat) and owes its popularity to low-cost production and the inexistence of any cultural or religious prohibition to its consumption. Chicken production is even more important in rural areas with economies based on subsistence agriculture. Additionally, besides being a source of food, in some regions of the globe, the chicken has been also used for cultural, religious, and entertainment proposes (Lawler, 2014).

The initiation of molecular genetic studies in the early 1990s has answered many questions regarding the origin, dispersal, and genetic diversity of many modern domestic chickens. It is now widely accepted that the red junglefowl (*Gallus gallus*) from jungles in South and Southeast Asia is considered the most probable ancestor of the domestic chicken (Fumihito et al., 1994; Fumihito et al., 1996). Historical and archaeological sources point to early domestication of the chicken, around 5,400 BC (West and Zhou, 1988; Underhill, 1997), although recent work on ancient DNA (aDNA) suggests northern China as the earliest chicken domestication site, around 8,000 BC (Xiang et al., 2014). Also, several recent genetic studies based on the mitochondrial DNA (mtDNA) variation have suggested the additional contributions of the red junglefowl from the Indian Subcontinent, South and East of China, Thailand, Myanmar, and Indonesia (e.g., see for more detail Liu et al., 2006; Miao et al., 2013).

The history of domestic animals in South America is similar to the rest of the "new world," in which the majority of the livestock species have been introduced by European colonizers from the 15^th^ century onwards. Although the indigenous guinea pig and the South American camelid species have been always considered a South American domestication, some authors, mostly based on archaeological evidence (Carter, 1971; Fitzpatrick and Callaghan, 2009; Rami´rez-Aliaga, 2010), have been arguing for a pre-Colombian introduction of the chicken in SA. Recently, the sequencing of the region of the mitochondrial genome from a Chilean bone dated from Ca. 1,304 to 1,424 AD suggested a pre-Columbian origin of the South American chicken (Storey et al., 2007). However, this work was contested by other authors (Gongora et al., 2008) as the mtDNA haplotype found at this site, and on which the authors argued as evidence of a Pacific origin of chicken in SA, belongs to a ubiquitous haplogroup (E) that can be found in chicken from all over the world. More recently, a study on the contemporary mtDNA diversity of several South American populations have found that although the Iberian Peninsula (European) chicken might have been the main source of the modern South American chicken, it also identified the presence of a genetic component in the Easter Island chickens that cannot be attributed to the introduction of chickens from Europe (through the Iberian Peninsula), and which is phylogenetically closer to the Southeast Asia populations (Luzuriaga-Neira et al., 2017).

Throughout time, successive waves of European colonizers have brought to South America their chicken stocks from their places of origin. With the intensification of chicken production in the twentieth century, new and highly selected and specialized breeds (e.g., egg-layers, broilers) have been created (Crawford, 1990), which have been spread worldwide at a much faster pace. However, the introgression of these highly selected and performant lineages of chicken into the local breeds has been impeded by the lower capacity of adaptation to most of the environmental conditions (e.g., temperature, parasites, predators). Most of the gene flow from the highly selected lineages has been made through F1s, in which a high performant lineage is crossed with a locally adapted breed.

In the last decade, access to next-generation sequencing (NGS) has permitted the development of more cost-effective and efficient techniques to measure variation at a genome-wide scale. NGS has permitted major advances in demographic parameters estimation as well as on the identification of genes underlying adaptation and production traits, and this in combination with phenotype data can accelerate breeding in plants and animals (e.g., review by Daetwyler et al., 2013). Thus, genome-wide variation studies can not only identify genomic regions underpinning the adaptation of certain populations to extreme environments (e.g., Zhang et al., 2016) as well as help conserving these regions while improving the productive performances of the local breeds (Thornton, 2010; Kristensen et al., 2015).

In this study, we used RADseq to scan and genotype hundreds of thousands of single-nucleotide polymorphism (SNPs) throughout the genome to characterize six SA local chicken populations from Bolivia, Brazil, Colombia, Chile (continental and Easter Island), Ecuador, and Peru. As cock-fighting has an important socio-cultural role in South America in the last centuries (Finsterbusch, 1990; Lawler, 2014), this region possesses a large number of gamefowls that have been bred separately from the others for many generations. Like the rest of the local populations, information on the origin and genetic structure of this population is very limited or unknown and for this reason we have included samples representing this population and three other populations representing old (Iberian Peninsula population) and two contemporary sources [a cosmopolitan meat production breed (broiler) and cosmopolitan egg-layers (Isa Brown)] that might have contributed for the current genetic architecture of the current South American local populations.

## Materials and Methods

### Tissue Sampling and DNA Extraction

Approximately 2 mm^2^ of the comb of 145 local domestic chickens were collected from six SA local populations representing: Bolivia (N = 6), Brazil (N = 4), Chile ((N = 35; 21 Mainland + 14 Easter Island), Colombia (N = 17), Ecuador (N = 16), Peru (N = 17), and gamefowl (N = 14). Individuals representing local Iberian Peninsula chicken (N = 17) as well as individuals representing commercial egg layers (N = 5; Isa Brown endproducts) and broiler (N = 15) were also sampled. Samples were stored in 95% ethanol at -20°C.

Genomic DNA was extracted using a JetQuick™ Tissue DNA Spin Kit (Genomed, GmbH) and quantified using a Qubit Fluorimeter (Thermo Fisher Scientific). RADseq sequencing libraries were prepared using the eight base-pair recognition site restriction enzyme *SbfI* (New England Biolabs, cat.# R3642L) using a new RAD protocol (Ali et al., 2016). In brief, DNA was normalized to 5 ng/µl and 10 µl of each sample was arrayed into a well in a 96-well plate. The DNA was cut using the eight base-pair recognition site restriction enzyme *SbfI* (New England Biolabs, cat.# R3642L). After cleavage, unique barcodes were ligated on and the samples were pooled, sheared in a Bioruptor NGS (Diagenode, Belgium), and used as input for NEBNext Ultra DNA Library Prep Kit for Illumina (New England Biolabs, USA). The libraries were sequenced on an Ilumina Hiseq 2500 using paired end 100 bp reads.

#### Data Analysis

We demultiplexed the libraries filtering solely the reads having a full barcode match and a partial restriction site match. Sequences were aligned to the Galgal4 Chicken Genome assembly (International Chicken Genome Sequencing Consortium, 2004), using the BWA algorithm (Li and Durbin, 2009), with the default parameters. Ambiguously mapped and/or clonal sequences were removed using the filters for proper pairs and PCR duplicates included in the SAMtools package (Li et al., 2009). The consensus sequences were constructed and the Binary sequence/Alignment Map format files (BAM) indexed using the same software package. To avoid bias caused by variable sequencing depth, we created subsampled BAM files using the random sampling option from SAMtools. We chose 180,000 alignments from each BAM file for the subsampled set. Genotype calls were performed using ANGSD (Korneliussen et al., 2014) with a minimum map quality score (minMapQ) and a minimum base quality score (minQ) of 20. For the variant calls, we used the SAMtools genotype likelihood model (Li, 2011) and selected sites present in at least 50% of the samples (minInd). To verify the performance of our SNP calling method, we have searched the public databases (the National Center for Biotechnology Information NCBI, dbSNP database, available at https://ftp.ncbi.nih.gov/snp/organisms/archive/chicken_9031/) for matches between our variants and those already identified in genome-wide studies. SNP annotation was performed using the SnpEff 3.0 program (Cingolani et al., 2012), using the galGal4 genome version as the reference.

#### Genetic Diversity

The two most common indexes of molecular genetic variation (θ)—mean pairwise differences between sequences (π; Tajima, 1989) and Watterson segregating sites (S; Watterson, 1975)—were calculated using thetaStat (ANGSD). Pairwise weighted F_ST_ windows were used to measure genetic differentiation between populations (Weir and Cockerham, 1984) using the VCFtools program (Danecek et al., 2011). Additionally, for estimating the genetic relationships between the potential population sources—i.e., samples representing Iberian Peninsula, broiler, egg-layer, South American gamefowl populations—and the South American chicken populations, we have also performed variance analyses (one-way ANOVA model) by comparing each pair of populations as a factor and the weighted F_ST_ value (per 50 kb sliding window) for the same pair of populations as the variable. Averages, standard error, and plots were generated using the R software (R Core Team, 2013).

**Figure 1:**
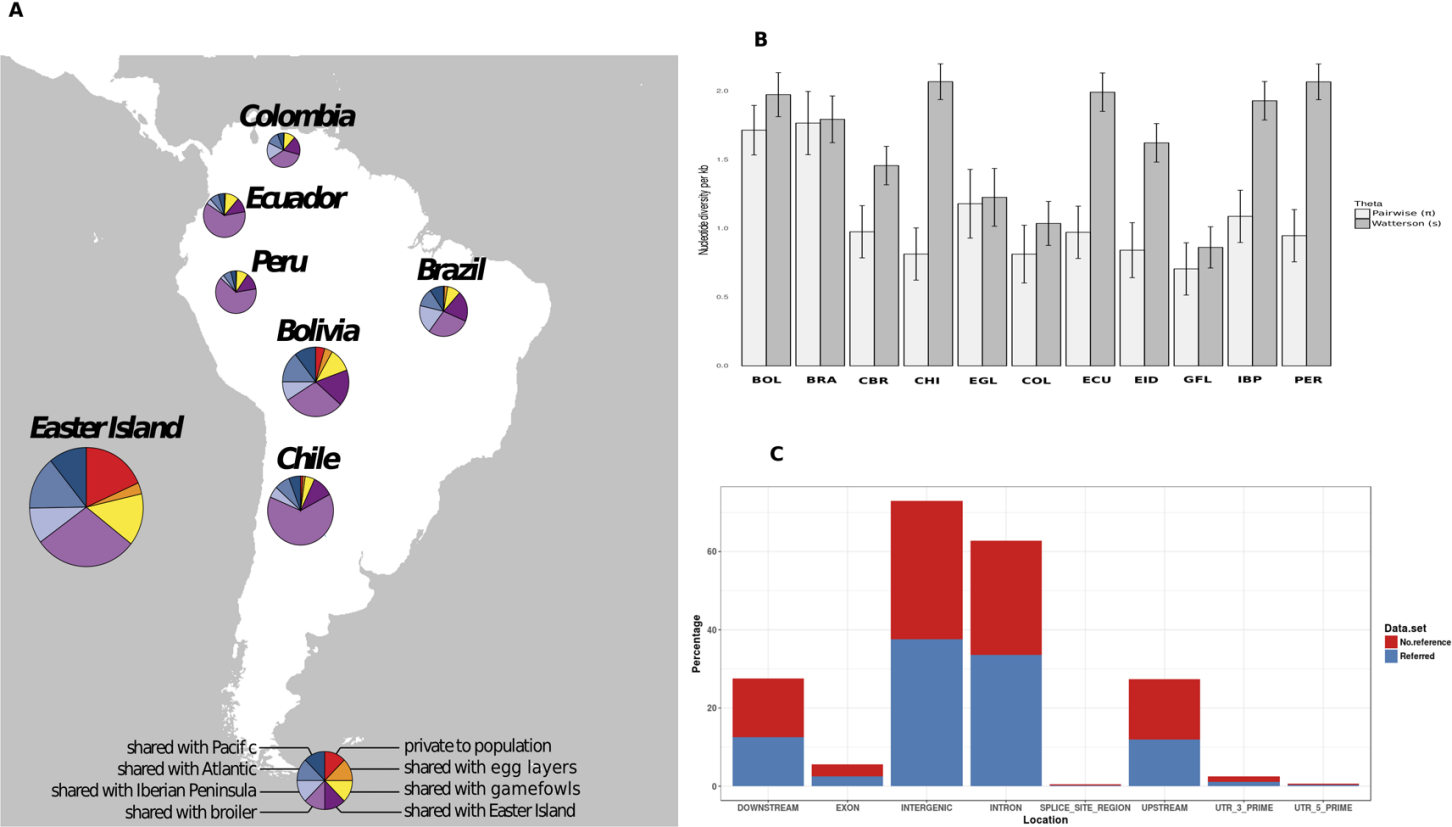
**(A)** Shared polymorphic variants within the South American chicken populations. Pie charts are divided into eight slices as is described in the figure legend at the bottom. The size of the circle is proportional to the number of the variants. **(B)** Bar plots of the mean estimate values of nucleotide diversity with the line corresponding to the mean standard error. Bolivia (BOL), Brazil (BRA), Broiler (CBR), Chile (CHI), Colombia (COL), Ecuador (ECU), Easter Island (EID), Gamefowl (GFL), Iberian Peninsula (IBP), Egg-layer (EGL), Peru (PER). **(C)** Variant effect location, colored areas are proportional to the percentage of previously reported (blue) and first time reported (red) variants.

To count the number of shared SNPs among South American chicken populations, we created Variant Call Format Files (VCF) for four groups of samples according to their geographical location. One group, composed by the individuals from South American countries located at Pacific fringe (Ecuador, Peru, Chile, and Bolivia), another group formed by individuals from the Atlantic fringe (Colombia and Brazil), and the potential source populations were kept in two separated groups. The number of shared variants between the groups was determined using the module vcf-compare included also in the VCFtools software, which conducts simple comparisons between VCF files. Venn diagrams (Caminsky et al., 2016; Feichtinger et al., 2016) were used to visualize private/shared variants per group. Those variants were then represented in pie charts (**Figure 1A**) representing variants in different categories: i) shared between the Pacific and Atlantic groups, ii) shared with any of the possible source populations (Egg layer, Broiler, gamefowl or Iberian Peninsula), and iii) unique to a group. Only variants displaying a ≥5% frequency per population were considered.

### Population Structure and Genetic Relationships

The *r* *^2^* parameter was estimated to identify SNPs in linkage disequilibrium (LD) using the software PLINK v1.9 (Purcell and Chang, 2015) for 50 kb sliding windows, over a phased file excluding SNPs with allele frequencies <0.05 and an *r* *^2^* > 0.5. A second filter was applied to remove all SNPs that significantly deviated from the expected neutrality. For this, we have used a Bayesian Fst-outliers based method that identifies loci, which the F_ST_ significantly depart from the average (F_ST_-outlier) (BayeScan v.2.1; Foll and Gaggiotti, 2008). After removal of all significantly linked SNPs, the dataset was phased using Beagle v3.3.2 (Browning and Browning, 2007).

The population structure and the pairwise genetic relationship between individuals from different populations were investigated using a principal component analysis (PCA) implemented in the ngsTools package (Fumagalli et al., 2014) and the resulting principal components (PCs) were plotted using the R script provided at the package website (available at https://github.com/mfumagalli/ngsPopGen/tree/master/scripts). The method implemented takes into account the genotype uncertainty and uses the output of the analyses performed in ANGSD to identify the polymorphic sites (SNP_pval 1x10^-6^), estimate the major and minor alleles (doMajorMinor 1), and infer the minimum allele frequencies (doMaf 2). Finally, we only retained loci with a minor allele frequency of <0.05 (minMaf). The posterior genotype probabilities were calculated with uniform *a priori* (doPost 2). The covariance matrix between individuals was calculated weighting each genotype for its posterior probability (Fumagalli et al., 2014).

To explore the relatedness among the chicken populations, we used the admixture model implemented in NGSadmix (Skotte et al., 2013). This method uses the genotype likelihood, taking into account the uncertainty of the genotype callings typical of the low-sequencing depth methods (Foote et al., 2016). For this analysis, we used the genotypes likelihoods determined in ANGSD and used the same set of filters as in previous analysis to avoid bias caused by outliers or linked loci. Several runs were done varying the number of K populations from 3 to 5; to extend this analysis, we have constructed a pie plot chart calculating the average contribution of all potential sources.

### The Origins of the South American Chicken Populations

Hypothetical ancestral admixture events among local South American chicken populations and the four possible population sources (Iberian Peninsula, egg layers, broiler, gamefowl) were assessed using TreeMix (Pickrell and Pritchard, 2012), which calculates a maximum likelihood population tree based on the allele frequencies. This method assigns an edge as a branch of the tree if it contributes with the majority of alleles to the descendant population; otherwise it is a migration edge. This process is performed in a stepwise likelihood mode to find the tree with the best fit for each admixture event (Pickrell and Pritchard, 2012). Here we used 117,962 autosomal phased SNPs, and the SNP dataset obtained from the genome resequencing of several red jungle fowls (Ulfah et al., 2016) as the outgroup.

The TreeMix results were also compared to those obtained using 3 Population Test (AdmixTools package; Patterson et al., 2012), which allows determining whether a population has inherited a mixture of ancestries (Reich et al., 2009). This method is similar to the *f3* (A, B, C), and when significantly negative values of the *f3* statistic are obtained, it implies that population A is admixed. Finally, ROLLOFF software (Patterson et al., 2012) was used to estimate the time of the admixture event. This method used the decay of the linkage admixture disequilibrium to approximate the time of admixture (Moorjani et al., 2011). In our case, the populations from the Iberian Peninsula and gamefowl were used as potential source populations and the South American populations as the admixed populations. The TreeMix results were used to select source populations to be tested in the 3 Population Test. As before, we divided the South American populations into two groups (Atlantic and Pacific).

## Results

### Genetic Diversity

Around 91% of our set of 122,801 nuclear SNPs matched with others already reported at dbSNP NCBI database. The majority of the identified variants were located in intergenic or intronic regions (**Figure 1**), from which approximately 60% were located across the nine macro chromosomes. On average, we roughly observed one SNP for every 8,900 bases (0.122 SNPs per kb).

Regarding the South American continental populations, the lowest number of private variants was observed in the Chilean continental populations, while the highest value was obtained in the Bolivian population. When grouping populations according to their geographic locations in South America (Atlantic and Pacific), all the populations showed a higher number of variants shared with the Pacific group, ranging from 108 in Peru up to 750 in Chile. In the Pacific group, the lowest number of private variants was found in Peru (19) and was highest in the Bolivian population (105). The Atlantic façade populations had a higher number of unique variants compared with the Pacific, with the maximum found in Brazil (113). The number of variants shared between the South American chicken and the egg layer was lower (between 1 and 46) than the number of variants shared with the broilers (between 17 and 124), the Iberian Peninsula (between 29 and 169), and the gamefowl (between 32 and 130). Individually, the Easter Island population displayed the highest values in terms of private and shared variants. A deeper analysis showed that 643 SNPs were exclusively found in the Easter Island population; 106 were shared only with egg layers, 367 only with broilers, 487 shared with gamefowl, 504 shared with the Iberian Peninsula, and 1,024 and 345 shared with the Pacific and the Atlantic South American groups, respectively (**Figure 1A**). The population diversity theta parameters (θ_S_ and θπ) estimated per 1,000 bp window attained the lowest values (θ_S_ and θπ) in the gamefowl population, and the Chile local chicken population showed the highest values for θ_S_ and the Brazilian and Bolivian population the highest values for θπ (**Figure 1B**).

### Population Structure and Genetic Relationships

**Figure 2:**
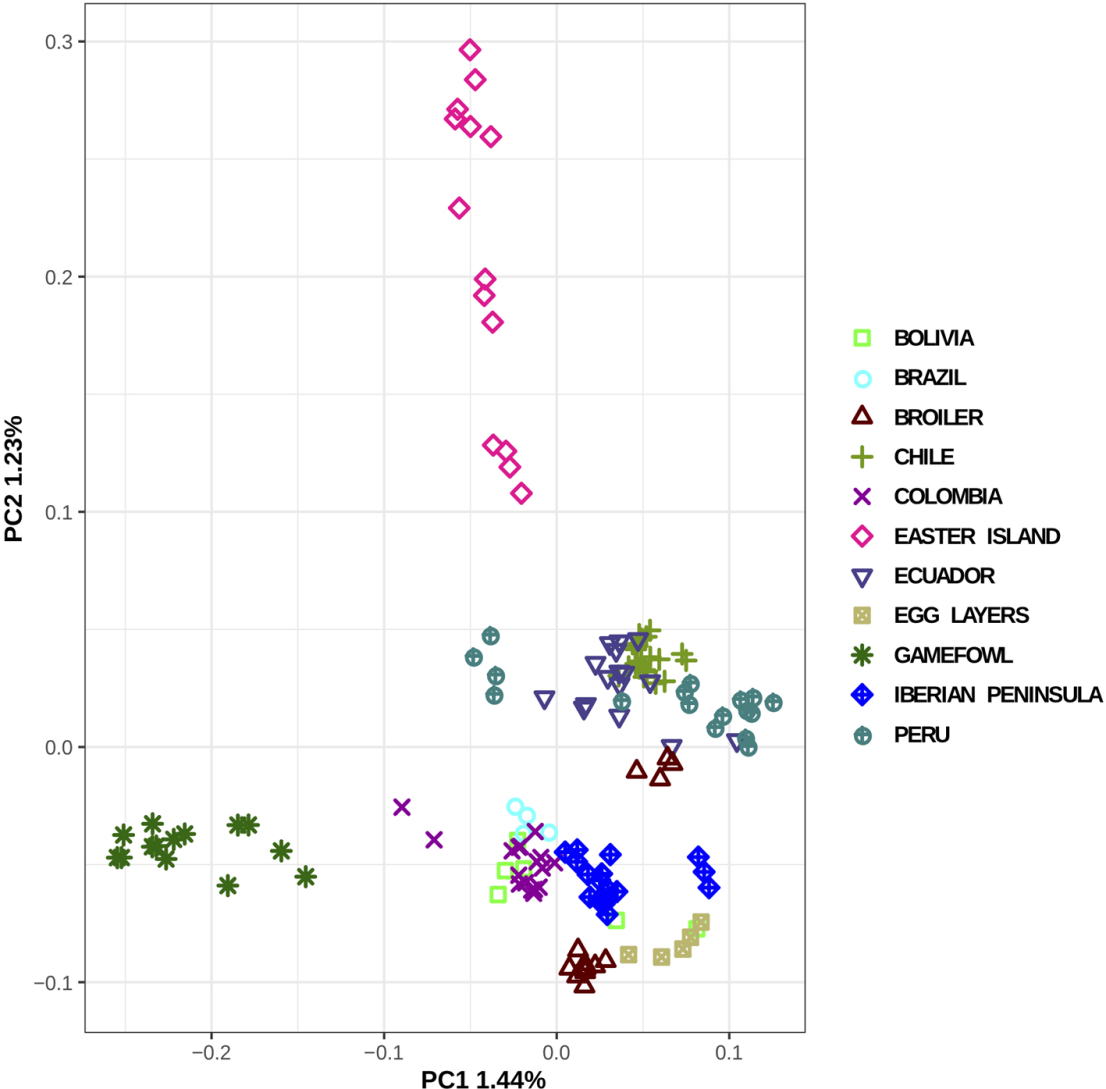
Principal component analysis of the local South American populations and putative genetic material sources.

Regarding the population structure and genetic relationships, the most remarkable finding revealed in the PCA plot (**Figure 2**) was the separation between the gamefowl and all the other South American chicken obtained in PC1, whereas PC2 separates Easter Island individuals from all the others. Another separation, although less evident, was the formation of two groups of populations, one containing all countries located in the SA Pacific façade (Ecuador, Peru, and Chile) and the other constituted by Brazil, Colombia, Bolivia, and Iberian Peninsula chicken. We have noticed a slightly higher tendency of the commercial breeds and Iberian population to cluster closer to the South America Atlantic group, whereas the Pacific group is genetically closer to the Easter Island than it is from the Iberian population.

Regarding the pairwise differentiation between the all analyzed populations (**Figure S1**), the gamefowl was the most differentiated population, with F_ST_ values ranging from 11% (Colombia) to 28% (egg layer). All the remaining populations showed lower differentiation levels ranging between 1% between Brazil and Bolivia and 17% between egg layer and Easter Island populations. A one-way ANOVA and Tukey’s *post hoc* analysis of the weighted F_ST_ estimates (50 kb sliding windows) showed that the differentiation between South America and the hypothesized population sources (Iberian Peninsula, broiler, egg-layer, gamefowl) is highly significant (*P* < 0.001). When ranking the potential source populations according to their degree of differentiation from the SA, the Iberian Peninsula showed the lowest differentiation (F_ST_ = 0.014), followed by the egg layer (F_ST_ = 0.039) and the broiler (F_ST_ = 0.056), and the gamefowl displayed the highest value (F_ST_ = 0.1) (**Figure S1**).

**Figure 3:**
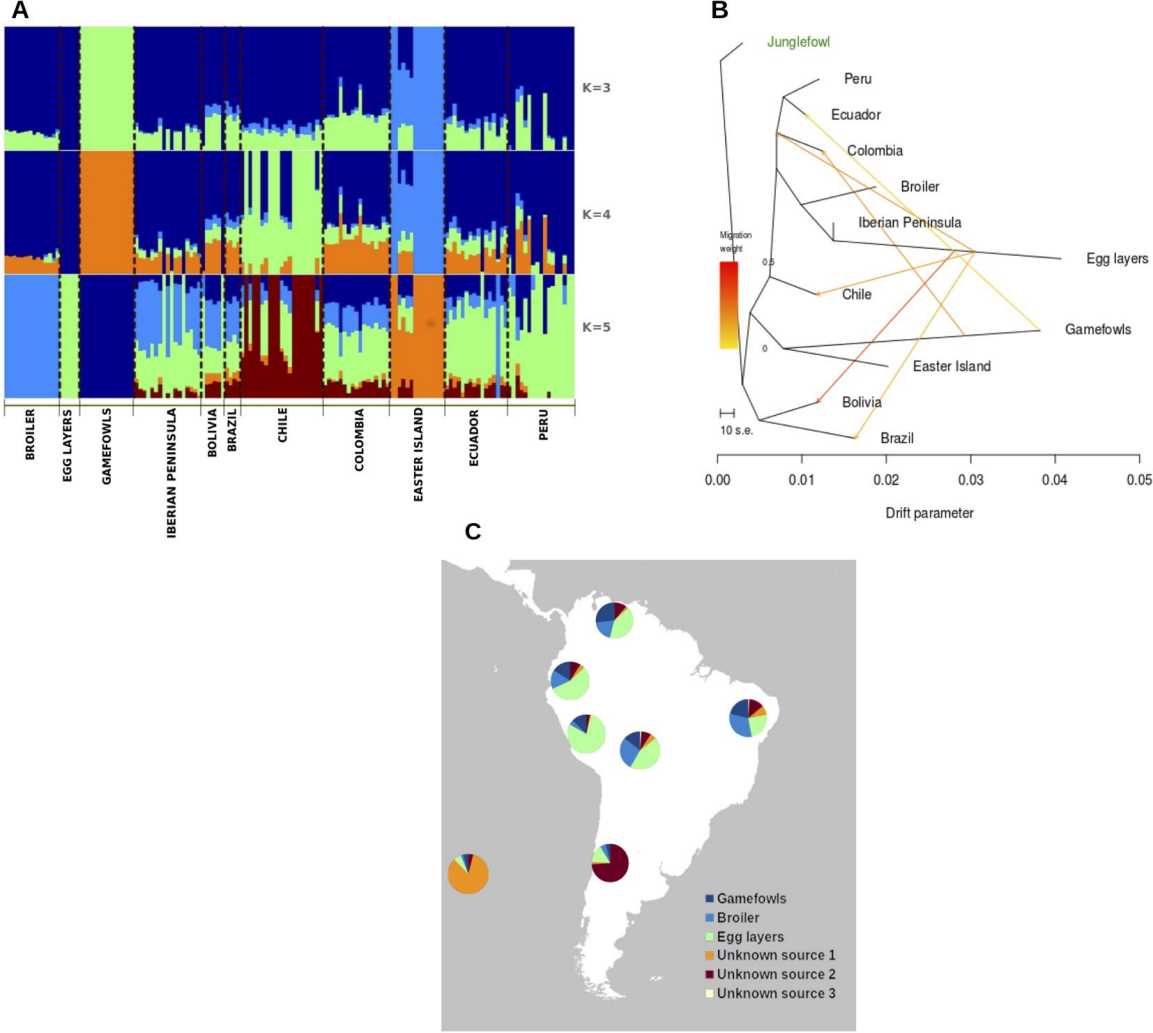
**(A)** Individual ancestry proportion of each of the South American chicken samples and putative genetic material sources conditional on the number of genetic clusters (k = 3-5). **(B)** TreeMix analyses of the genetic relationships between South American chicken and potential source populations. **(C)** Putative genetic material sources conditional on the number of genetic clusters.

The Bayesian clustering analysis performed with NGSadmix was consistent with the PCA results. The relatively closely related group formed by all South American populations depicted by the PCA is also confirmed by plotting the admixture analysis results (**Figure 3**). Here, we observe a certain degree of admixture between all the South American chickens and the influence of the commercial egg layers and broiler lineages as well as the gamefowl in the contemporary South American chicken. The Easter Island population displays a different admixture pattern in which a specific (non-shared) genetic background is very pronounced. Moreover, the admixture plot shows that in the Easter Island population, the most frequent genetic component is represented, although at a very small frequency, at the continental South American populations.

### The Origins of the South American Populations

As the previous analysis pointed to a large influence of commercial breeds in the South American chicken, we have quantified this influence using TreeMix analyses. The obtained phylogenetic tree reflects the divergence patterns among the different chicken populations (**Figure 3**) and depicts the large influence of the egg layer in the South American chicken (**Figures S2**, **S3**).

The *f*_3_-statistics analysis, through *3-population test*, to confirm the introgression events identified using the TreeMix method, returned significant values for the combinations *f*_3_ (Pacific; egg layer, gamefowl) and *f*_3_ (Atlantic; egg layers, gamefowl). For the Pacific–egg layer–gamefowl combination, the calculated values were *f*_3__=_ -0.0017, Z = -12.44 and for the Atlantic–egg layers–gamefowl combination, calculated values were *f*_3__=_ -0.0017, Z = -16.599 (Table S1).

Finally, to quantify the contribution of each potential source, we have calculated the average values based on the NGSadmix results (**Figure 3C**). With the exception of Chile, the local South American chicken populations were highly influenced by commercial chicken breeds, in which the egg-layers average admixture level ranges between 44% and 79%, while the broiler had a lower influence with an average admixture level ranging between 16% and 32%, and the gamefowl contribution ranges from 4% to 27% with the lowest in Chile and the highest to Colombia. Chile and Easter Island populations show different admixture patterns relative to the other populations with a high percentage of contributions from unknown sources. Interestingly, the results from the roll-off analyzes that are dated to be the most influential migratory events from around 70 ± 10 generations ago, which represents between 70 and 35 years considering a 1-year or 2-year generation interval, respectively.

## Discussion

### Genetic Diversity

The commercial and relatively accessible high-density SNP array for the chicken became the most common tool used in genomic studies recently. However, the use of this pre-ascertained SNP panel distorts population genetic inferences on local livestock populations, as the sample sizes and the highly selected populations in which SNPs were discovered pose significant biases (Albrechtsen et al., 2010; Lachance and Tishkoff, 2013). Here, we used reduced representation library sequencing, in this case, RADseq, to interrogate a medium-high number of SNPs (122,801). The comparison of this set with those SNPs identified in the NCBI dbSNP database revealed that 91% of our SNPs match with others previously identified and 97% of them are located in intergenic or intronic regions, showing great potential to be used in genetic diversity studies.

The summary statistics of genetic variation using two theta estimators (θπ and θs) showed similar diversity per population (**Figure 1B**). The gamefowl proved to be an exception to this as they showed the lowest values and can be explained as the result of the inbreeding practices used to swiftly fix desired traits (García, 1997). The very similar values obtained for the two parameters (θs, θπ) in Brazil and Bolivia populations are better explained by the sample size effect (Korneliussen et al., 2013), as the sampling for both populations was substantially smaller than for the other South American populations. On the other hand, the different values displayed between the two theta parameters, with the θs showing higher values than θπ, at the remaining populations (e.g., CHI, PER, ECU, and IBP), can be explained by differences in the proportion of alleles segregating at intermediate frequencies. It is known that the θπ algorithm ascribes more weight to alleles segregating at intermediate frequencies, while θs weights all categories equal (Korneliussen et al., 2013), and thus populations showing a lower number of alleles with intermediate frequencies will result in smaller θπ values.

The patterns of the genetic variants shared among the different populations also provide insights about the continental South American chicken population diversity. Interestingly, the Easter Island population is the one displaying the highest number of unique variants (643), and this can be interpreted as the result of its different demographic history and/or different population origins. The high number of unique alleles could be explained by the different origins of the chicken introduced on this island across time (Luzuriaga-Neira et al., 2017). Alternatively, the high number of shared variants between this population and the other continental South American chickens can be explained by a source-sink metapopulation process (e.g., Gaggiotti, 1996). The occurrence of this phenomenon can simultaneously explain the occurrence of a high number of private variants (sink) and shared variants (source) as the result of different migration events from SA continent that have arrived at this island since at least 1772 (Wilhelm, 1957).

### Population Structure and Genetic Relationships

The PCA plot (**Figure 2**) constructed with all individuals shows that the individuals belonging to the gamefowl and Easter Island populations are relatively well separated from the remaining populations. Curiously, despite the low differentiation between the remaining continental South American populations, the PCA divides them into two groups, which might be related with whether its geographic location is on the Atlantic *façade* (Brazil, Columbia) or the Pacific *façade* (Peru, Chile, Ecuador).

The large differentiation indicated by F_ST_ estimates between the gamefowl and all the other South American populations (Table S2) is not very surprising. The different breeding objectives (i.e., behavior) and the observed low levels of diversity are the two most probable causes of this high differentiation regarding the other South American populations. Indeed, the admixture analysis shows the absence of influence from the other tested breeds in the gamefowl (**Figure 3A**) but shows some influence of this population in the other populations. This might indicate that the different breeding goal of this population, regarding the rest, has prevented its crossing with the commercial chicken breeds, particularly with the commercial egg-layer breed, as is evident in the other South American populations.

The Easter Island population is a very interesting example, as despite being the most divergent from the other populations, it is also the one in which its individuals are relatively more dispersed. The PCA grouping of the individuals (**Figure 2**) is a relatively good method to detect the coancestry relationship among individuals from the same population. It is expected that two individuals closely related would be closer to each other, but the Easter Island population has individuals that are considerably more distant from the others of their own population than relatively other individuals from other populations (e.g., Peru). In fact, this pattern is usually associated with different migration events (Fumagalli et al., 2013; Schraiber and Akey, 2015), and in this case, may indicate the influence of the chicken populations from the SA Pacific fringe in the Easter Island population. The higher differentiation is displayed by both Easter Island and the gamefowl populations, whereas the small differentiation amidst South American chicken populations and between these and the commercial breeds suggests differential gene-flow rates as the main driver of the extant South American chicken population structure. 

The post-Columbian human migration events and the subsequent spread of people from the coastal areas to the interior become particularly massive at the end of the nineteenth century and might have led to multiple introductions of chicken from different populations. The quantification of the admixture proportion for each of the studied populations and a large number of migration edges needed to add (13) to explain most of the variance (99.8%) depicted by the phylogram (**Figure S2**) demonstrates that those populations have had a constant flux of foreign genes.

### The Origins of the South American Chicken Populations

It has been hypothesized that European and Asian chickens were introduced in SA after 1500 (Storey et al., 2011); nevertheless, the modern introductions have been less described. However, we found that a single source population (Iberian Peninsula) could not explain the diversity displayed by the South American chicken suggesting a different demographic history for the South American chicken populations, opening the possibility of a multiple origin scenario. The poultry industrialization that started after World War II resulted in the globalization of massive industrial production and dispersal, leading to extensive crossbreeding between individuals from few highly selected and cosmopolitan chicken varieties (egg-layers, broilers) with local varieties, which have taken place in SA. Remarkably, the *roll off* admixture analysis detected signs of a strong introgression in SA population dating between 35 and 70 years ago, which is concordant with the worldwide expansion of poultry industry based on highly productive chicken lineages. If this is correct, then the current SA local chicken accumulates the legacy of the older chicken introduced with those modern highly selected varieties.

In Ecuador, Peru and, Colombia, cock-fighting is a popular part of their culture and local recreation activities (Finsterbusch, 1990). However, the origin of the SA gamefowl is poorly known, with many anecdotal reports linking their introduction with the arrival of Spanish and Portuguese colonizers who may have brought these birds from their colonies in South and Southeast Asia, where cock-fighting is a very ancient tradition (Lawler, 2014). Here, we could not identify the potential source population, but the TreeMix tree positions it at the same branch with the Easter Island population (**Figure 3B**), which might be indicative of a common origin of these two populations. Although the Easter Island chicken may have their roots linked to the Polynesian people expansion throughout the South Pacific (Wilhelm, 1957; Fitzpatrick and Callaghan, 2009), which have arrived at Easter Island around 1,200 A.D. (Hunt and Lipo, 2006), its genetic proximity with the SA continental gamefowl can be explained by the fact that both populations were not crossed with cosmopolitan breeds and therefore remain closer to the ancestral population that originated them. Moreover, if this is true, then these populations may represent the genomes of the first chicken that were introduced in this part of the world, which have been replaced in other populations by uncontrolled crosses between local and newly selected chicken cosmopolitan populations (broiler and egg-layers) that were developed during the intensification of poultry production. Indeed, the admixture levels obtained in this study point for a replacement of the local genomes of the older local chicken populations that were taken from the Iberian Peninsula to South America five centuries ago.

## Data Availability Statement

The datasets generated for this study can be found in the BioProject database: https://www.ncbi.nlm.nih.gov/bioproject/573756 (Accession: PRJNA573756).

## Ethics Statement

Standard techniques were used to collect very small piece of tissue from each animal, by local veterinary trained personnel. The procedure was reviewed and approved by CIBIO-University of Porto Committee of ethics.

## Author Contributions

AB-P, AL-N, GV-R, and FC-C conceived the study. AL-N, SO’R and AB-P drafted the manuscript. AL-N, LP-P, and MM participated in the data analysis. AL-N and SO’R did the laboratory work. AL-N, AU-N, GE-S, J A-P, MV and MR-Q did the sampling. AB-P and MRM supervised the study. All the authors read and approved the manuscript.

## Funding

This work was supported by funds from the project NORTE-01-0145-FEDER-000007, from the Norte Portugal Regional Operational Program (NORTE2020), under the PORTUGAL 2020 Partnership Agreement, through the European Regional Development Fund (ERDF). AL-N was supported by a doctoral grant from SENESCYT, LP-P was a recipient of a postdoctoral grant from the Portuguese Science Foundation (FCT) (SFRH/BPD/94518/2013), and AB-P was a recipient of an IF contract from the FCT.

## Conflict of Interest

The authors declare that the research was conducted in the absence of any commercial or financial relationships that could be construed as a potential conflict of interest.
