# Supplementary material for: The Local South American Chicken Populations Are a Melting-Pot of Genomic Diversity
Source: Front Genet. 2019 Nov 19;10:1172. doi: 10.3389/fgene.2019.01172 (PMC6877731; doi:10.3389/fgene.2019.01172)
Supplement: Supplementary file 1 [file DataSheet_1.pdf]

Supplementary Material

South American chicken populations are a melting pot of genomic diversity

Luzuriaga-Neira, A.; Pérez-Pardal, L.; O'Rourke, S.M.; Villacís-Rivas G.; Cueva-Castillo F.; Escudero-Sánchez G.; Aguirre Pabón J.C.; Ulloa-Nuñez A.; Rubilar-Quezada M.; Vallinoto, M.; Miller, M.R.; Beja-Pereira A.

Supplementary Tables

Table S1- Results of the 3-population test

Source 1	Source 2	Target	*f3*	Std. Err.	Z
Gamefowl	Egg-layer	Pacific group	-0.1771	0.0014	-12.414
Gamefowl	Egg-layer	Atlantic group	-0.0237	0.0014	-16.599

Table S2 – Weir & Cockerham's F_{ST} distance matrix between the studied populations

	Bolivia	Brazil	Chile	Colombia	Easter Island	Ecuador	Egg layers	Iberian Peninsula	Broiler	Peru	Gamefowl
**Bolivia**	0.000										
**Brazil**	0.010	0.000									
**Chile**	0.050	0.047	0.000								
**Colombia**	0.043	0.039	0.036	0.000							
**Easter Island**	0.106	0.105	0.082	0.082	0.000						
**Ecuador**	0.026	0.028	0.022	0.023	0.071	0.000					
**Egg-layer**	0.087	0.149	0.083	0.086	0.168	0.067	0.000				
**Iberian Peninsula**	0.030	0.035	0.031	0.030	0.086	0.015	0.059	0.000			
**Broiler**	0.072	0.076	0.060	0.063	0.116	0.046	0.130	0.038	0.000		
**Peru**	0.035	0.042	0.029	0.031	0.032	0.006	0.074	0.020	0.053	0.000	
**Gamefowl**	0.188	0.180	0.139	0.112	0.158	0.123	0.287	0.150	0.179	0.139	0.000

Supplementary Figures

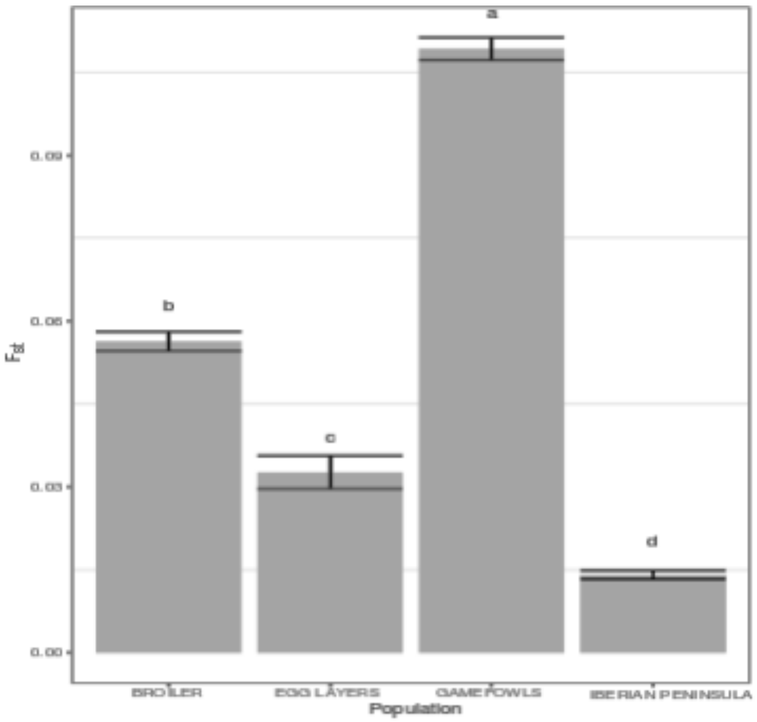

Figure S1. Bar plot depicting the significant differences detected between weighted F_{ST} values ($p < 0.2x10^{-16}$) calculated between South American chicken and potential source populations. The differences were analyzed using one-way ANOVA model, letters where assigned according the Tukey's post-hoc test, bars represent the mean standard error.

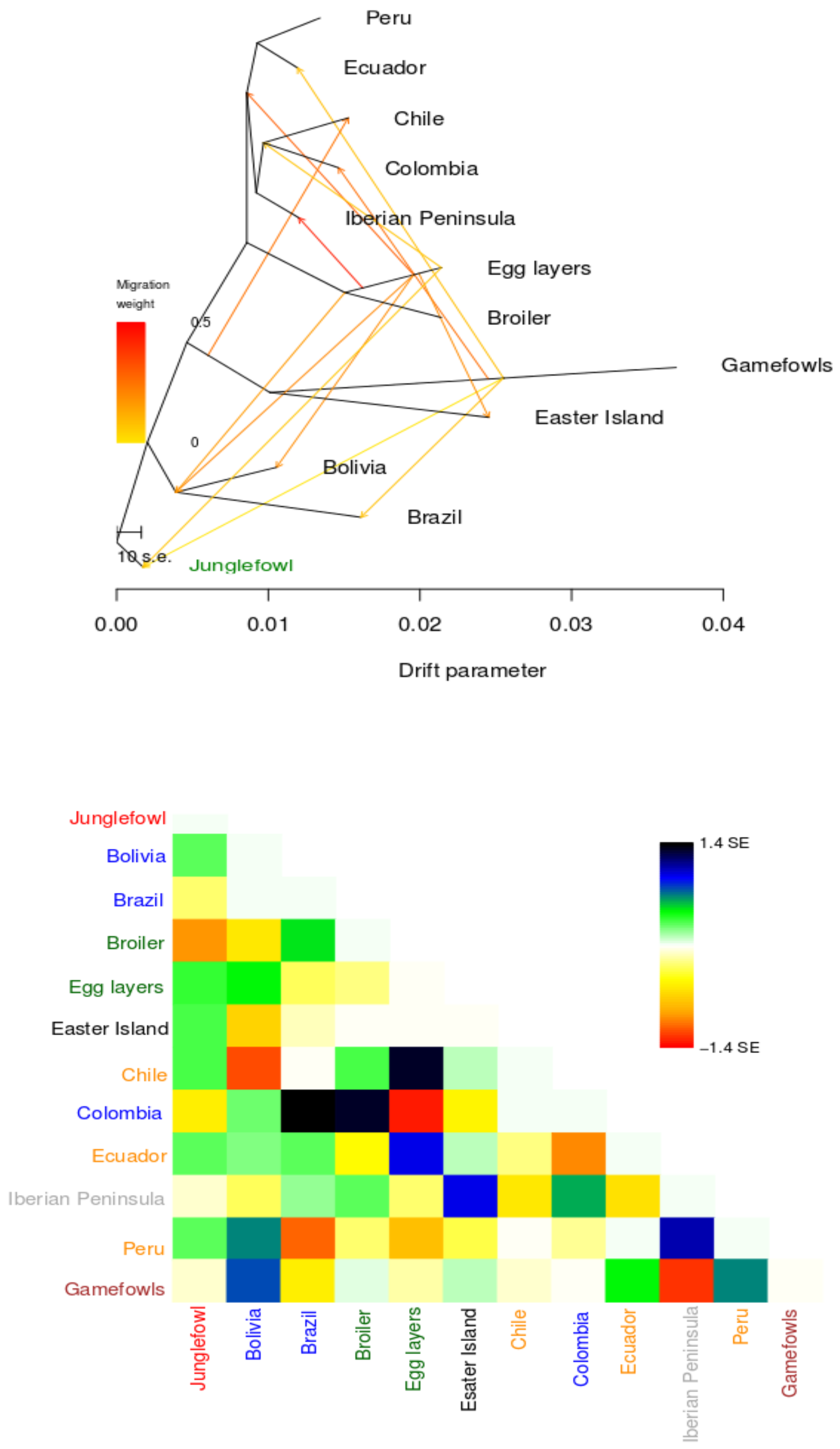

Figure S2. Phylogenetic network model and its residuals fit considering 13 migration edges.

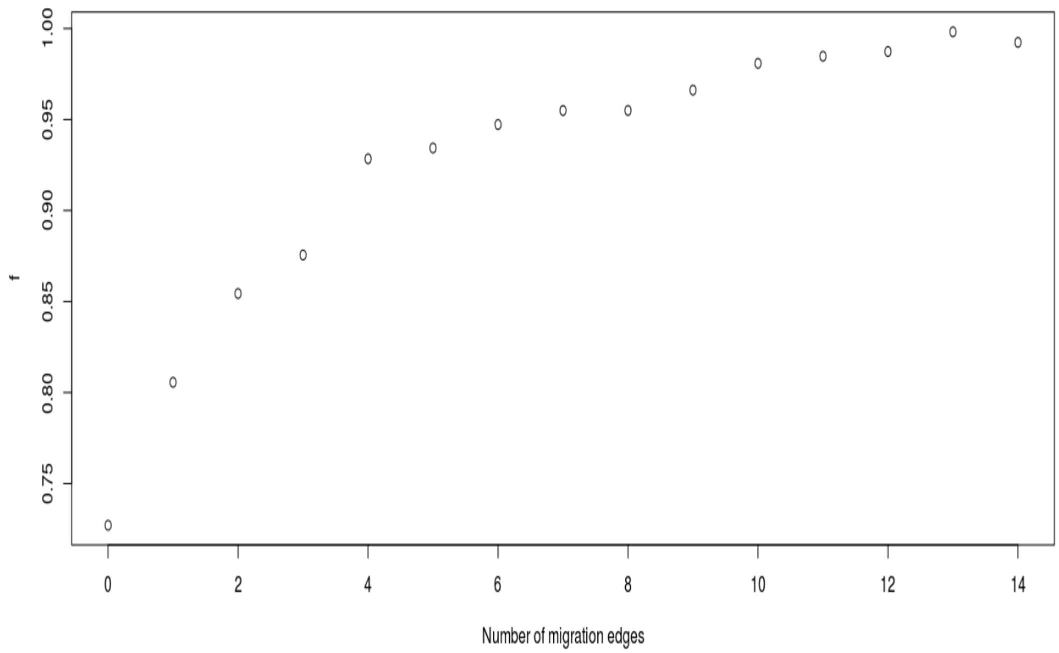

Figure S3. Scatter plot representing the fit of the genetic relatedness between populations according to the explained variance for each model from 0-13 edges.
